# Supplementary material for: The Tryptophan Index Is Associated with Risk of Ischemic Stroke: A Community-Based Nested Case–Control Study
Source: Nutrients. 2024 May 21;16(11):1544. doi: 10.3390/nu16111544 (PMC11174068; doi:10.3390/nu16111544)
Supplement: Supplementary file 1 [file nutrients-16-01544-s001.zip › nutrients-3000099-supplementary.pdf]

Supplementary Table S1 Demographic and clinical characteristics by quartiles of tryptophan index at baseline (n=642).

	Tryptophan index (×100)					*P* trend
	Total	Q1 (<11.2)	Q2 (11.3, 12.5)	Q3 (12.6, 13.7)	Q4 (>13.8)	
Age (years)	69.6 (63.4, 75.1)	71.5 (63.6, 76.8)	69.2 (63.0, 75.3)	71.7 (65.3, 74.9)	69.5 (63.2, 75.2)	0.010
Male (%)	284 (44.2)	95 (52.2)	67 (39.6)	67 (45.9)	55 (37.9)	0.032
BMI (kg/m^2)	23.5 (21.4, 25.8)	23.5 (21.4, 26.2)	24.3 (21.8, 26.3)	22.9 (20.6, 25.0)	23.6 (21.2, 25.5)	0.041
Current smoking (%)	163 (25.4)	51 (28.0)	39 (23.1)	37 (25.3)	36 (24.8)	0.60
Physical activity (MET-h/d)	21.3 (12.6, 34.8)	17.6 (12.0, 30.8)	21.4 (14.1, 33.5)	24.5 (12.9, 40.4)	22.2 (12.4, 34.2)	0.11
Educational attainment (%)						0.37
0 year	310 (48.4)	81 (44.5)	84 (50.0)	65 (44.5)	80 (55.2)	
1–5 years	242 (37.8)	73 (40.1)	58 (34.5)	63 (43.2)	48 (33.1)	
≥6 years	89 (13.9)	28 (15.4)	26 (15.5)	18 (12.3)	17 (11.7)	
TC (mmol/L)	4.88 (4.25, 5.58)	4.85 (4.28, 5.51)	4.92 (4.25, 5.63)	4.96 (4.22, 5.64)	4.91 (4.26, 5.40)	0.63
TG (mmol/L)	1.27 (0.94, 1.79)	1.35 (0.95, 1.95)	1.36 (0.95, 2.04)	1.19 (0.99, 1.56)	1.22 (0.90, 1.61)	0.017
HDL-C (mmol/L)	1.44 (1.19, 1.69)	1.36 (1.13, 1.64)	1.44 (1.17, 1.64)	1.54 (1.22, 1.73)	1.52 (1.30, 1.82)	<0.001
Fasting glucose (mmol/L)	5.46 (5.01, 6.10)	5.83 (5.08, 6.57)	5.43 (5.01, 6.10)	5.41 (4.98, 5.86)	5.26 (4.99, 5.77)	<0.001
Diabetes (%)	96 (15.0)	44 (24.2)	25 (14.8)	19 (13.0)	8 (5.52)	<0.001
Hypertension (%)	474 (73.8)	133 (73.1)	127 (75.2)	103 (70.6)	111 (76.6)	0.72
eGFR (ml/min/1.73 m^2)	84.8 (73.0, 92.5)	83.8 (69.9, 90.3)	84.8 (73.2, 92.7)	83.1 (73.0, 90.0)	88.7 (77.8, 95.0)	<0.001
Hyperlipidemia (%)	169 (26.3)	56 (30.8)	53 (31.4)	34 (23.3)	26 (17.9)	0.003

P trend across quartiles of tryptophan index and characteristics of baseline were examined by Jonckheere-Terpstra test for continuous variables, Cochran-Armitage trend test for binary categorical variables, and Cochran-Mantel-Haenszel test for other categorical variables. Abbreviations: BMI, body mass index; eGFR, estimated glomerular filtration rate; HDL-C, high-density lipoprotein cholesterol; MET, metabolic equivalent; TC, total cholesterol; TG, triglyceride.

Supplementary Table S2 Incidence rate ratio (IRRs) and 95% confidence intervals (CI) for ischemic risk by conditional logistic regression models.

	Model 1	Model 2	Model 3
	IRR (95% CI)	IRR (95% CI)	IRR (95% CI)
**Tyrosine (µmol/L)**			
Q1 (<78.1)	1.00 (Ref)	1.00 (Ref)	1.00 (Ref)
Q2 (78.2, 90.1)	0.74 (0.47, 1.15)	0.69 (0.43, 1.09)	0.60 (0.37, 0.98)
Q3 (90.2, 107)	0.71 (0.47, 1.08)	0.65 (0.42, 1.01)	0.67 (0.42, 1.05)
Q4 (>108)	0.81 (0.52, 1.24)	0.75 (0.48, 1.17)	0.70 (0.44, 1.23)
P for trend	0.25	0.15	0.15
Continuous	0.91 (0.78, 1.07)	0.89 (0.75, 1.05)	0.89 (0.75, 1.05)
P value	0.24	0.15	0.17
**Valine (µmol/L)**			
Q1 (<235)	1.00 (Ref)	1.00 (Ref)	1.00 (Ref)
Q2 (236, 265)	1.09 (0.70, 1.70)	1.14 (0.72, 1.79)	1.18 (0.73, 1.91)
Q3 (266, 298)	1.06 (0.70, 1.61)	1.10 (0.72, 1.69)	1.11 (0.70, 1.76)
Q4 (>299)	1.27 (0.83, 1.94)	1.25 (0.80, 1.95)	1.07 (0.65, 1.74)
P for trend	0.32	0.38	0.85
Continuous	1.11 (0.95, 1.30)	1.10 (0.93, 1.30)	1.04 (0.87, 1.25)
P value	0.18	0.25	0.68
**Phenylalanine (µmol/L)**			
Q1 (<61.9)	1.00 (Ref)	1.00 (Ref)	1.00 (Ref)
Q2 (62.0, 69.4)	0.95 (0.62, 1.47)	0.95 (0.62, 1.48)	0.81 (0.51, 1.31)
Q3 (69.5, 80.0)	0.89 (0.57, 1.39)	0.87 (0.55, 1.38)	0.71 (0.43, 1.15)
Q4 (>80.1)	0.96 (0.60, 1.52)	0.89 (0.55, 1.43)	0.73 (0.44, 1.22)
P for trend	0.79	0.55	0.19
Continuous	1.02 (0.86, 1.19)	0.98 (0.83, 1.16)	0.92 (0.77, 1.10)
P value	0.84	0.82	0.37
**Isoleucine (µmol/L)**			
Q1 (<71.7)	1.00 (Ref)	1.00 (Ref)	1.00 (Ref)
Q2 (71.8, 85.5)	1.16 (0.73, 1.85)	1.26 (0.78, 2.04)	1.14 (0.68, 1.92)
Q3 (85.6, 103)	1.12 (0.72, 1.76)	1.22 (0.77, 1.93)	1.17 (0.71, 1.94)
Q4 (>104)	1.51 (0.97, 2.36)	1.55 (0.98, 2.44)	1.39 (0.84, 2.30)
P for trend	0.08	0.08	0.21
Continuous	1.19 (1.02, 1.39)	1.20 (1.02, 1.41)	1.18 (0.98, 1.41)
P value	0.027	0.026	0.08
**Leucine (µmol/L)**			
Q1 (<99.7)	1.00 (Ref)	1.00 (Ref)	1.00 (Ref)
Q2 (99.8, 115)	0.89 (0.57, 1.38)	0.87 (0.55, 1.36)	0.91 (0.56, 1.46)
Q3 (116, 131)	0.85 (0.54, 1.32)	0.82 (0.52, 1.29)	0.75 (0.46, 1.21)
Q4 (>132)	1.23 (0.79, 1.91)	1.16 (0.74, 1.83)	1.00 (0.61, 1.63)
P for trend	0.43	0.59	0.82
Continuous	1.12 (0.95, 1.32)	1.09 (0.92, 1.30)	1.03 (0.85, 1.23)
P value	0.18	0.30	0.78

**Total CAAs (μmol/L)**			
Q1 (<565)	1.00 (Ref)	1.00 (Ref)	1.00 (Ref)
Q2 (566, 629)	1.16 (0.75, 1.81)	1.21 (0.77, 1.91)	1.39 (0.85, 2.25)
Q3 (630, 699)	1.20 (0.79, 1.84)	1.23 (0.80, 1.89)	1.21 (0.76, 1.94)
Q4 (>700)	1.24 (0.80, 1.93)	1.21 (0.76, 1.90)	1.05 (0.64, 1.73)
P for trend	0.34	0.44	0.95
Continuous	1.11 (0.94, 1.29)	1.09 (0.92, 1.28)	1.03 (0.86, 1.23)
P value	0.21	0.33	0.78

Model 1: unadjusted model, conditioning on individual case set; Model 2: adjusted for BMI (continuous), current smoking (yes, or no), educational attainment (0 year, 1-5 years, or ≥6 years), and physical activity (by quartiles), conditioning on individual case set; Model 3: further adjusted for family history of stroke (yes, or no), hypertension (yes, or no), diabetes (yes, or no), hyperlipidemia (yes, or no), and eGFR (continuous). Natural logarithmic (log) transformation was conducted to achieve approximately normal distributions for continuous CAAs. Abbreviations: BMI, body mass index; eGFR, estimated glomerular filtration rate.
